# Pregnancy Among Women with Kidney Transplantation: A 20-Years Single-Center Registry

**DOI:** 10.1055/s-0039-1688834

**Published:** 2019-06-16

**Authors:** Stephanye Mariano, Jose Paulo de Siqueira Guida, Marcos Vinicius de Sousa, Mary Angela Parpinelli, Fernanda Garanhani Surita, Marilda Mazzali, Maria Laura Costa

**Affiliations:** 1 Departament of Tocogynecology, School of Medical Sciences, Universidade Estadual de Campinas, SP, Brazil; 2 Renal Transplant Research Laboratory, Renal Transplant Unit, Division of Nephrology, School of Medical Sciences, Universidade Estadual de Campinas, SP, Campinas, Brazil

**Keywords:** kidney transplantation, pregnancy, high-risk, hypertension, premature birth, dialysis, transplante renal, gravidez de alto risco, hipertensão, parto prematuro, diálise

## Abstract

**Objective** To assess maternal and perinatal outcomes in pregnancies after kidney transplantation in a tertiary center in Brazil.

**Methods** Retrospective cohort of pregnancies in women with kidney transplantation at the Universidade Estadual de Campinas, from January 1995 until December 2017. Medical charts were reviewed, and maternal and perinatal outcomes were described as means and frequencies. Renal function and blood pressure were evaluated during pregnancy and postpartum.

**Results** A total of 22 women had at least 1 pregnancy during the considered time interval, and 3 of them had > 1 pregnancy, totalizing 25 pregnancies. The mean age at transplantation was of 24.6 ± 4.2 years old, and the mean time interval until pregnancy was of 67.8 ± 46.3 months. The most frequent complication during pregnancy was hypertension, which affected 11 (64.7%) women. The gestational age at delivery was 34.7 ± 4 weeks, and 47% of these pregnancies were preterm (< 37 weeks). A total of 88.2% of the women delivered by cesarean section. Renal function, measured by serum creatinine, remained stable during pregnancy, and the systolic blood pressure increased significantly, while the diastolic blood pressure did not differ during pregnancy.

**Conclusion** Pregnancy after kidney transplantation is a rare event. Pre-eclampsia and prematurity were frequent complications, and cesarean section rates were very high. A specialized antenatal and postpartum care with a multiprofessional approach and continuous monitoring of graft function are essential for the early diagnosis of complications and improved outcomes.

## Abstract (pt)

**Resumo:**

**Objetivo** Avaliar os resultados maternos e perinatais de gestações em mulheres transplantadas renais em um centro terciário no Brasil.

**Métodos** Coorte retrospectiva de gestações entre mulheres transplantadas renais na Universidade Estadual de Campinas, de Janeiro de 1995 a Dezembro de 2017. Os prontuários médicos foram revisados, e os resultados maternos e perinatais foram descritos como médias e frequências. A função renal e a pressão arterial foram avaliadas durante a gravidez e o puerpério.

**Resultados** Um total de 22 mulheres tiveram ao menos 1 gravidez durante o período avaliado, e 3 delas tiveram > 1 gestação, totalizado 25 gestações. A idade média no momento do transplante foi 24.6 ± 4.2 anos, e o tempo médio de intervalo até a gravidez foi de 67.8 ± 46.3 meses. A complicação mais frequente durante a gravidez foi a hipertensão, que acometeu 11 (64.7%) mulheres. A idade gestacional no parto foi de 34.7 ± 4 semanas, e 47% das gestações encerraram-se prematuramente (< 37 semanas). Um total de 88.2% das gestações terminou com uma cesárea. A função renal, avaliada pela creatinina sérica, permaneceu estável durante a gravidez, enquanto a pressão arterial sistólica aumentou significativamente. A pressão arterial diastólica não diferiu ao longo dos períodos avaliados.

**Conclusão** Gestação após o transplante renal é um evento raro. Pré-eclâmpsia e prematuridade foram as complicações mais frequentes, e as taxas de cesárea foram muito altas. O cuidado multiprofissional no pré-natal e no puerpério e a constante monitoração da função do enxerto são fundamentais para diagnosticar precocemente complicações e melhorar os resultados.

## Introduction

The rise of hypertension and diabetes in recent years, along with other less prevalent causes, has increased the occurrence of chronic kidney disease (CKD), a progressive loss of renal function, classified in five stages, according to the glomerular filtration rate.^1^

The prevalence of CKD in Brazil is of 1.5% and, currently, 3 to 6 million people live with this disease in the country, the majority of them untreated. In addition, there are ∼ 120 thousand patients on dialysis,^2^ and > 92 thousand people already underwent renal transplantation in the country.^3^

In advanced stages, CKD may impair fertility due to anovulatory cycles caused by hypothalamic-pituitary-axis dysfunction.^4^ Fertility can be restored after renal transplantation with the improvement of kidney function.^5^

Pregnancy after kidney transplant is associated with increased maternal and fetal complications.^5^ Obstetrics complications, such as hypertension, gestational diabetes mellitus, fetal growth restriction and preterm deliveries were observed in many previous studies.^5^ ^6^ ^7^ One fifth of pregnancies ends as abortion; however after the 1^st^ trimester, most pregnancies present adequate development.^8^

There is a lack of studies about renal and obstetrics outcomes in pregnancy after kidney transplantation. Knowledge about the topic is limited to a small number of cohort studies, most of them performed in high-income countries.^9^

The aim of the present study is to evaluate maternal and perinatal outcomes after kidney transplantation in a referral center in the southeast region of Brazil.

## Methods

This is a retrospective cohort of all women followed at the Renal Transplant Unit of the Universidade Estadual de Campinas from January 1995 to December 2017. In the present analysis, we selected women who got pregnant after transplantation and had their antenatal care at the Women's Hospital of the same University. Both units are public tertiary health services that cover ∼ 5 million people in the southeast region of Brazil. The medical charts of the women included in the present analysis were reviewed by researchers using a preapproved data collection tool. We collected data on sociodemographic characteristics, underlying cause of CKD, age at transplantation, time between the surgery and pregnancy, immunosuppressive therapy on pregnancy onset, and also graft loss after pregnancy. We also obtained data on obstetric characteristics, such as duration of pregnancy, occurrence of clinical complications (hypertensive diseases, diabetes), route of delivery, and postpartum infection. The perinatal outcomes evaluated were birthweight and Apgar Score at birth.

### Statistical Analyses

Data were stored in Windows Excel 7 (Microsoft Corporation, Redmond, WA, USA) and analyzed with Epi Info 7 (Centers for Disease Control and Prevention, Atlanta, GA, USA). Data are presented in frequencies or means and standard deviation (SD).

### Ethical Approval

The ethics board from the Universidade Estadual de Campinas approved the research protocol (CAAE 64490017.6.0000.5404), and individual consent was waived since this is a medical chart review. The present manuscript was written according to the strengthening the reporting of observational studies in epidemiology (STROBE) recommendations.^10^

## Results

**Fig. 1:**
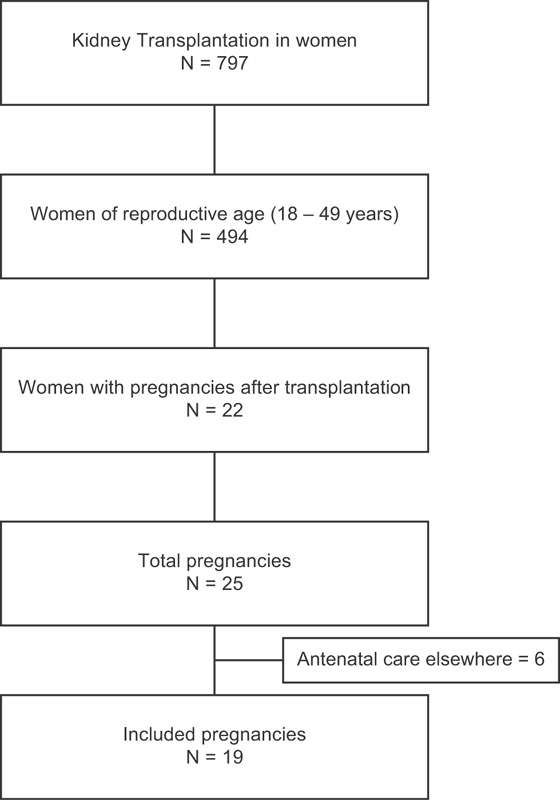
Flowchart of inclusion.

A total of 797 women with kidney transplantation were identified; of those, 494 were of reproductive age (18–49 years old) and had their medical charts reviewed. A total of 22 women had at least 1 pregnancy during the considered time interval, and 3 of them had > 1 pregnancy, totalizing 25 pregnancies. Of those, six did not have their antenatal care at the Universidade Estadual de Campinas, and 19 were included in the present analysis. Among the included pregnancies, 2 had a spontaneous abortion, and 17 were further considered for maternal and perinatal outcomes. Fig. 1 presents a flowchart of the cases included in the present study.

The mean age at transplantation was of 25.0 ± 4.0 years old, and the mean time interval until pregnancy was of 70.6 ± 45.3 months. The majority of women were white, and most of them lost their kidney function due to hypertension. Azathioprine, prednisone, and cyclosporine were the most used immunosuppressive drugs before pregnancy. The majority of women had their 1^st^ pregnancy after the transplantation. Table 1 presents these data.

**Table 1:** Sociodemographic characteristics of pregnant women with previous kidney transplant

Variable	
*n*	19
Age at transplantation (years old, mean ± SD)	25.0 ± 4.0
Underlying cause of chronic kdney disease (*n*, %)	
Hypertension	6 (31.6)
Chronic glomerulonephritis	3 (15.8)
Chronic pyelonephritis	3 (15.8)
Drug nephrotoxicity	1 (5.3)
Unknown	6 (31.6)
Skin color (n, %)	
White	16 (84.2)
Non-white	3 (15.8)
Prepregnancy immunosuppressive drug (n, %)	
Azathioprine	18 (94.7)
Prednisone	16 (94.1)
Cyclosporine	14 (82.3)
Tacrolimus	1 (5.9)
Prepregnancy weight (kilograms, mean ± SD)	64.5 ± 12.7
Parity (n, %)	
1	9 (47.4)
2	4 (21.0)
≥ 3	6 (31.6)
Transplantation-to-pregnancy interval (months, mean ± SD)	70.6 ± 45.3

Abbreviation: SD, standard deviation.

The most frequent complication during pregnancy was hypertension, which affected 11 (64.7%) of the women followed-up in this analysis. Five (29.4%) of them had pre-eclampsia, and 2 (11.8%) with severe features (they received magnesium sulfate). Four (23.5%) women were admitted to the intensive care unit (ICU), and 1 (5.9%) needed dialysis temporarily during the postpartum period. Only 2 (11.8%) of them had postpartum infection. Fetal growth restriction affected 4 (23.5%) of these pregnancies. Table 2 shows these data. Among the women included, only 1 presented graft failure, 7 years after the pregnancy.

**Table 2:** Pregnancy complications of pregnant women with previous kidney transplantation

Variable	*n* (%)
	17^a^
Complications during pregnancy (n, %)	
Hypertension^b^	11 (64.7)
Pre-eclampsia	5 (29.4)
Use of magnesium sulfate (severe features)	2 (11.8)
Gestational diabetes	1 (5.9)
Dialysis^c^	1 (5.9)
Intensive care unit admission	4 (23.5)
Fetal growth restriction	4 (23.5)
Postpartum infection	2 (11.8)

A: Two pregnancies that ended as 1^st^ trimester miscarriage were excluded. B: Hypertension included chronic hypertension, gestational hypertension, and preeclampsia. C: In the postpartum period; no cases were reported during pregnancy.

**Fig. 2:**
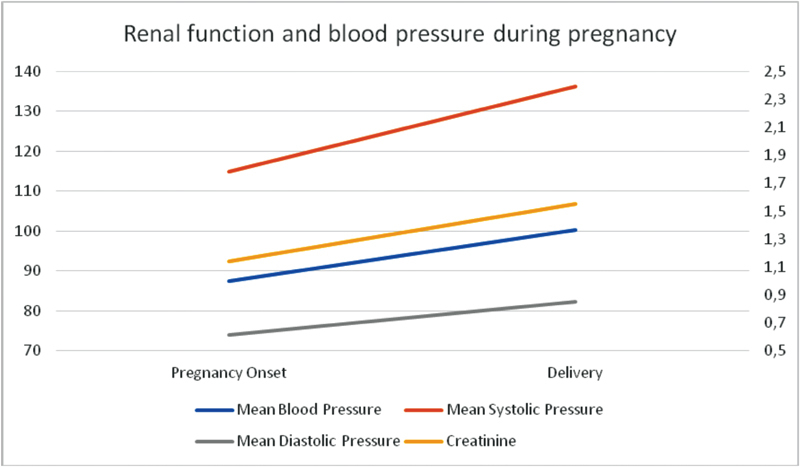
Renal function and blood pressure during pregnancy.

Table 3 presents data regarding delivery. Gestational age at delivery was 34.7 ± 4 weeks, and 47% of those pregnancies were preterm (< 37 weeks). A total of 88.2% of the women delivered by cesarean section, and maternal reasons justified the majority of the cesarean sections performed. The mean birthweight at delivery was 2,339 ± 820 g, and only 3 (17.6%) babies were small for gestational age, and only 1 newborn had an Apgar score at 5 minute < 7. Almost all (14; 82.3%) of the women were using a contraceptive method postpartum; among them, definitive sterilization was chosen by 6 women, while long-term methods (levonorgestrel intrauterine system and copper intrauterine device) and short-term methods (oral contraceptive or injectable progesterone) were chosen by 4 women each.

**Table 3:** Perinatal outcomes and postpartum contraception in women with pregnancy after kidney transplantation

Variable	
*n*	17^a^
**Mean maternal age at delivery (years old, mean ± SD)**	30.5 ± 4.0
**Gestational age at delivery (weeks, mean ± SD)**	34.7 ± 4.0
**Preterm delivery (n, %)**	8 (47.0)
< 34 weeks	4
≥ 34 weeks	4
**Delivery route (n, %)**	
Vaginal	2 (11.8)
Cesarean	15 (88.2)
Maternal indication^b^	10
Fetal indication^c^	5
**Birthweight at delivery (grams, mean ± SD)**	2339 ± 820
**5 minute Apgar score ≤7 (** ***n*** **, %)**	1 (5.9)
**Small-for-gestational age (** ***n*** **, %)**	3 (17.6)
**Postpartum contraception (** ***n*** **, %)**	14 (82.3)
Definitive sterilization	6 (35.3)
Levonorgestrel intrauterine system	3 (17.6)
Oral contraceptive	3 (17.6)
Copper intrauterine device	1 (5.9)
Injectable progesterone	1 (5.9)

Abbreviation: SD, standard deviation.

A: Two pregnancies that ended as 1^st^ trimester miscarriage were excluded. B: Maternal indications included: maternal choice, worsening of maternal conditions, unfavorable pelvis, cephalic-pelvic disproportion. C: Fetal indications included acute or chronic fetal distress.

Renal function, measured by serum creatinine, remained stable during pregnancy, considering a first measurement during admission to antenatal care and another in the end of pregnancy or delivery (Fig. 2). Systolic blood pressure upgraded significantly during pregnancy, while diastolic blood pressure did not differ in initial and delivery measures (Table 4).

**Table 4:** Renal function and blood pressure during pregnancy

	Pregnancy – 1^st^ Evaluation^A^	Admission for birth	*p-value*
Creatinine (mg/dL)	1.14 ± 0.84	1.55 ± 1.37	0.3
Mean blood pressure (mmHg)	87.5 ± 13.7	100.3 ± 16.0	0.02
Mean systolic pressure (mmHg)	114.8 ± 16.1	136.2 ± 21.9	< 0.01
Mean diastolic pressure (mmHg)	73.9 ± 13.1	82.4 ± 14.1	0.08

A: In the 1^st^ trimester.

## Discussion

Our study showed that pregnancies after kidney transplantation are rare events, with good perinatal and clinical outcomes. Hypertension was an important complication among the women followed in our study, with an important incidence of pre-eclampsia. This result may be due to the use of cyclosporin as a maintenance immunosuppressive therapy in the majority of the transplant recipients evaluated. Previous studies have shown a rate > between 60 and 70% of maternal complications such as pre-eclampsia and hypertension in renal transplant recipients receiving cyclosporin, similar to the rates observed in our study. In addition, hypertension was the main etiology of chronic kidney disease in our cohort, which could be related to the elevated incidence of hypertension and pre-eclampsia during the follow-up. The prolonged use or high dose of glucocorticoids could also be associated with the onset or with the worsening of hypertension in this series. The side effects of azathioprine, another immunosuppressive drug frequently used in our series, at doses used in kidney transplantation, include gastrointestinal intolerance, bone marrow suppression, liver function abnormalities, and infections, with no effect on blood pressure. Also, in this series, teratogenic drugs such as mycophenolate or mammalian target of rapamycin (mTOR) inhibitors were avoided or discontinued in fertile women who expressed the desire of pregnancy, and were replaced by azathioprine, as a transplant unit routine.^11^

Spontaneous abortion is a known concern among women who use immunosuppressant therapy due to the effect of the drugs used. In our cohort, only two abortions were reported, a result similar to those of other reports,^12^ ^13^ and we had no cases of fetal malformation, probably due to the adequacy of the therapeutic scheme adopted toward women planning pregnancy. The placental concentration of cyclosporin used to be between 5 and 10 times higher than that found in maternal blood, with the fetus blood level about half than that found in the mother. Because of this, cyclosporin does not lead to an increased risk for fetal malformations after exposure. Azathioprine is mutagenic, but its teratogenicity was demonstrated in animal studies at doses much higher than those used in kidney transplantation. Glucocorticoid teratogenicity was excluded by previous studies even for intravenous high doses.^11^ Currently, tacrolimus is the first-choice drug for immunosuppression after kidney transplantation; however, until 2004, cyclosporin was the first-line immunosuppressive drug in our service. Patients with stable allograft function were kept with that drug.^14^

Reproductive-aged women who received a kidney graft may desire pregnancy after the improvement of their quality of life and the normalization of their reproductive cycles, even knowing this may be a high-risk pregnancy.^15^ Medical morbidities, as many other social conditions, affects the reproductive cycle of women, including pregnancy, delivery and the postpartum period, and many of those are not modifiable conditions.^16^ Having a transplanted kidney is one of those conditions, and a multidisciplinary approach to pregnancy, including nephrologists and obstetricians, may improve the experience of the women during pregnancy and reduce obstetrical and clinical complications during pregnancy, with timely diagnoses and treatment.

Hypertensive disorders are a key concern during the antenatal care of women after kidney transplantation, since it can be a life-threatening condition during pregnancy and may affect allograft function. Pre-eclampsia was a common complication in our cohort, as it was in other previous reports.^12^ ^13^ ^17^ Prescribing low-dose aspirin and calcium supplementation, with an intensive surveillance of blood pressure has been the standardized follow-up of these women in our center. Also, we believe that a frequent assessment of the renal function using serum creatinine and 24-hour proteinuria is mandatory in these cases.

Our cesarean rates were high, mostly due to the worsening of maternal conditions. While another two Brazilian studies seem to have lower cesarean section rates,^17^ ^18^ a most recent European study presented rates similar to ours.^19^ Having previous kidney transplantation is not a reason to perform a cesarean section, and induction of vaginal delivery is possible in the majority of cases. Despite that, our perinatal outcomes were satisfactory, proving that close surveillance of these women, during pregnancy and delivery, is fundamental.

Counseling about contraception to these women is very important, and our data show that the majority used long-term methods postpartum. Definitive sterilization, after providing qualified information, may be an option; however, intrauterine reversible devices are also a safe intervention.^20^

We present here the experience of the institution in the last 20 years with kidney transplant and pregnancy. The total number of cases seems low; however, they are not, for a single center, and the results on maternal and perinatal outcomes are good, with an integrated multidisciplinary approach. Unfortunately, we do not have long-term follow-up postpartum or data on pregnancy desire and planning among transplantation cases.

## Conclusion

Pregnancy after kidney transplantation is a rare event. In our case series, the rates of spontaneous abortion were similar to those of the general population, the main complications were due to hypertensive disorders, with an important occurrence of prematurity and high rates of cesarean deliveries. Pregnancy did not seem to affect graft function; however, the increase in blood pressure may have long-term consequences and calls for continuous follow-up. A multidisciplinary team and regular evaluation of pregnancy and transplantation-related complications are the key for a good care.
